# Deformation Monitoring Systems for Hydroturbine Head-Cover Fastening Bolts in Hydroelectric Power Plants

**DOI:** 10.3390/s25082548

**Published:** 2025-04-17

**Authors:** Eddy Yujra Rivas, Alexander Vyacheslavov, Kirill V. Gogolinskiy, Kseniia Sapozhnikova, Roald Taymanov

**Affiliations:** 1 Department of Metrology, Instrumentation and Quality Management, Empress Catherine II Saint Petersburg Mining University, Saint Petersburg 199106, Russia; 2 Materials Science Department, Petersburg Nuclear Physics Institute, Gatchina 188300, Russia; 3 Laboratory for Metrological Maintenance of Computerized Sensors and Measuring Systems, D. I. Mendeleyev Institute for Metrology, Saint Petersburg 199005, Russia; k.v.s@vniim.ru (K.S.);

**Keywords:** Francis turbine, vibration, deformation, fastening bolts, monitoring system

## Abstract

**Highlights:**

**What are the main findings?**

- The synthesis and analysis of available data reveal a significant gap in research on monitoring systems for the deformation of hydroturbine head-cover fastening bolts.
- The analyzed monitoring systems do not guarantee their own operational integrity, including their metrological characteristics.

**What are the implications of the main findings?**

- The physical integrity of the fastening bolts on hydroturbine head-covers is not guaranteed, increasing the risk of accidents in hydroelectric power plants. Therefore, monitoring systems need to be improved.
- The reliability of the measurement results is not assured, which may thus increase errors in decision making. Metrological self-check should be introduced into monitoring systems.

**Abstract:**

This study investigates the reliability of Francis turbines and highlights the critical need for an improved deformation monitoring system for bolts that fasten a hydroturbine head-cover to its casing. During different operational stages of the hydraulic unit, such as start-up, partial load, and full load, the hydroturbine head-cover and its fastening bolts are subjected to static and cyclic loads. The loads generate vibrations and different deformations that must be monitored. Although various measuring instruments, such as vibration sensors and accelerometers, have been developed to monitor hydroturbine vibrations, only two systems—KM-Delta-8-CM and PTK KM-Delta—are currently applied to measure fastening bolt deformation. Furthermore, only one system, SKDS-SISH, was found to monitor the forces inducing this deformation. After analysis, it is evident that the described systems for monitoring the deformation of the fastening bolts do not guarantee the trustworthiness of the measuring sensors and there is a need for their improvement. The implementation of a self-checking function (including metrological features), the development of a digital twin of the sensor, and the application of technologies based on artificial intelligence could solve this problem.

## 1. Introduction

Hydroelectric power plants (HPPs) are essential to the generation of electricity that powers virtually every aspect of modern society, from lighting and domestic appliances to industry and critical infrastructure.

A considerable body of research has been dedicated to the discussion of prospects for the development of the electric power industry in different countries. The increase in electricity consumption is predicted [1,2], and the need for new hydropower plants is emphasized [3,4]. As indicated in [5], the capacity of HPPs in the world is 1170 GW, which makes them the main source of electricity. And despite the fact that research continues to improve the efficiency of electricity generation utilizing solar, wind, oil, and gas energy, the need for hydropower sources continues to grow, especially in the mining [6] and oil and gas industries [7,8].

On the other hand, environmental concerns play a significant role in electricity production. According to information from the International Hydropower Association (IHA) and the International Energy Agency (IEA), hydropower plants remain the largest source of renewable energy and are crucial for achieving net-zero greenhouse gas emissions, including carbon dioxide and other pollutants, by 2050 [1,9]. Therefore, it is essential to ensure the reliability of hydropower plants and to mitigate the negative impacts of more intensive operational modes on their hydraulic and mechanical components. This challenge is largely addressed by integrating measuring instruments into control systems, including those that perform monitoring.

A hydroelectric power plant predominantly utilizes radial–axial turbines, commonly referred to as Francis turbines. These facilities are equipped with two control systems. One control system manages direct operations related to energy generation, such as turbine control, safety measures, and protection protocols [10,11]. The other control system contributes to optimal resource management and compliance with regulatory standards [11]. The development and enhancement of these control systems are essential for ensuring the efficient and stable operation of HPPs, thereby preventing accidents.

**Figure 1:**
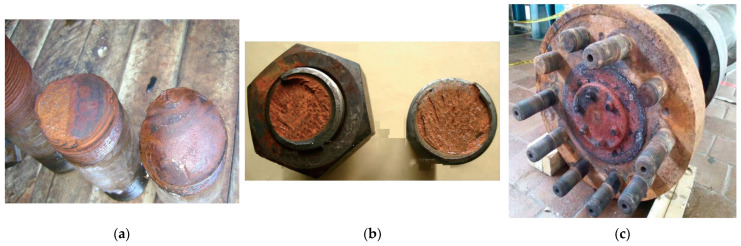
Failures of hydroturbine fastening bolts in several countries: (**a**) Russia [15]; (**b**) China [13]; (**c**) Colombia [14].

The disaster at the Sayano-Shushenskaya Hydroelectric Power Plant (SSHPP) in Russia in 2009 not only shocked the whole country but also prompted a review of monitoring and control systems at hydroelectric power plants worldwide. According to the official report on the causes of the accident presented by Rostekhnadzor [12], the tragedy at Unit 2 of the SSHPP occurred due to the failure of the fastening stud bolts (hereinafter referred to as bolts) on the hydroturbine head-cover (see Figure 1a). The failure was attributed to significant and prolonged vibrations; the operation of the hydroturbine under an undesirable combination of water pressure and power output; and human errors related to maintenance violations and a lack of timely decisions.

Article [13] mentions another fatal incident at a large HPP in China in 2016, which resulted from the failure of fastening bolts on the hydroturbine head-cover (see Figure 1b). The hydroturbine cover and rotor were lifted by high-pressure water.

In 2018, a study was presented on fatigue failures observed in three out of ten fastening bolts that connect the turbine shaft to the generator in an HPP in Colombia (see Figure 1c). The authors attributed the failures to cyclic loading conditions combined with environmental factors that may have accelerated wear and damage [14].

Considering these events, this article aims to analyze the current state of deformation monitoring systems in fastening bolts of Francis turbine head-covers, focusing on opportunities to enhance their effectiveness.

This review consists of several sections. Section 2 discusses the Materials and Methods. Section 3 describes the operational stages of a hydraulic unit. Section 4 analyzes the vibrations and deformations occurring in Francis turbines. Section 5 examines instruments for vibration measurements as well as methods for measuring deformations in Francis turbines. Section 6 presents the monitoring systems for hydroturbine head-cover fastening bolts at the SSHPP. Section 7 provides a discussion and analysis of this review. The Section 8 presents the conclusions drawn from this scientific review and suggests topics for future research.

## 2. Materials and Methods

To assess the current state of monitoring systems related to the fastening bolts of hydroturbine head-covers, research papers in this field, review articles, international standards, and technical specifications of measuring instruments were examined. This involved searching for articles in scientific literature databases (Scopus, Elibrary) and scientific journals published by MDPI, IoP, and Elsevier. The standards under consideration were sourced from materials published by ISO and Rosstandart.

The resolution of the figures was enhanced using Photoshop CS5 Portable 12.1.0.

The present study employed a theoretical research method aimed at understanding the condition of the monitoring systems for the fastening bolts installed on the hydroturbine head-cover through the analysis and synthesis of information obtained from documents and scientific articles.

## 3. Operational Stages of a Hydraulic Unit

A hydraulic unit (HU) refers to a complete system used in HPPs to convert the hydraulic energy of flowing or falling water into electrical energy. The HU typically includes the hydroturbine, electric generator, control systems, and auxiliary equipment. The operation of an HU comprises a sequence of coordinated stages that are intricately linked to the behavior of the hydroturbine head-cover. Below is a detailed description of the operational stages of an HU and the corresponding behavior of the hydroturbine head-cover.

### 3.1. Stages of Operation of a Hydraulic Unit

**Figure 2:**
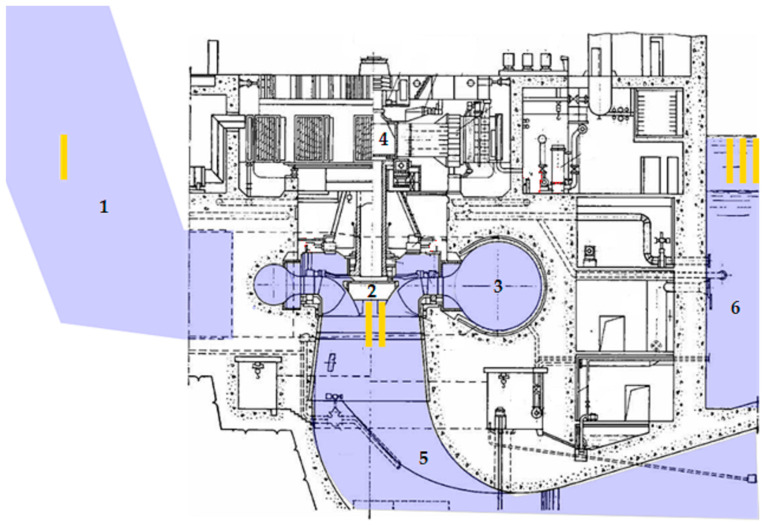
Cross-section view of a hydraulic unit installation [compiled by the authors]. 1—penstock; 2—hydroturbine; 3—spiral chamber; 4—generator; 5—draft tube; 6—downstream reservoir.

To explain the operational stages of the HU, a cross-section of an HPP was segmented into three distinct zones, as illustrated in Figure 2:

- Zone I—penstock. This zone connects the upstream reservoir, where a volume of water accumulates upstream, thereby generating potential energy. This potential energy is subsequently converted into electrical energy.
- Zone II—spiral chamber, hydroturbine, and generator. In this zone, pressurized water flows through the spiral chamber, where its potential energy is transformed into kinetic energy. As the water enters the engine room, it impinges on the blades of the hydroturbine, converting its kinetic energy into mechanical rotational energy. The hydroturbine axis is coupled to that of the generator, which, upon rotation, converts the rotational energy into medium- or high-voltage alternating current.
- Zone III—downstream reservoir. After relinquishing its energy, the water is discharged downstream of the HPP via a drainage channel.

Prior to initiating the HU, a series of preparatory steps are undertaken to ensure a safe and efficient startup. This preparatory stage, referred to as the pre-startup, involves the following sequential steps [16,17,18]:

1. Initial preparation—zones I, II, and III are completely drained. The HU is stationary, with neither the rotor nor the electric generator rotating. No electrical load is supplied, as the HU is disconnected from the electrical grid.
2. Downstream bypass valve opening—zones I, II, and III are filled to the downstream level. The HU remains stationary, with no rotation of the rotor or electric generator. No electrical load is supplied. Subsequently, the downstream gate is opened.
3. Upstream bypass valve opening—zone I is fully flooded to the upstream water level. The HU remains stationary, with no rotation of the rotor or electric generator. No electrical load is supplied. Following this, the upstream gate is opened.

The startup stage begins, comprising the following sequential steps:

4. HU unlocking—the hydroturbine blades are unlocked and opened. The HU remains stationary, with no rotation occurring. No electrical load is supplied to the grid.
5. Opening of downstream gates—the HU begins to rotate and accelerate to its operational speed. Despite reaching operational speed, no power is delivered to the grid.
6. Stabilization—the rotation frequency of the rotor stabilizes, entering a state commonly referred to as ‘no-load operation’. During this step, the HU continues to operate without transferring power to the grid.

Depending on the operating mode of the HU, two distinct operational stages are identified: partial load and full load operation. The transition to these stages involves the following critical step:

7. Grid connection—the HU has achieved its operational speed. It is then connected to the electrical grid, enabling the delivery of electrical power.

Upon the completion of its working cycle or when maintenance is required, the HU enters the shutdown stage. This stage involves the following sequential steps:

8. Grid disconnection—the HU is disconnected from the electrical grid, ceasing power delivery.
9. Closing of upstream gates—the water level begins to decrease. As a result, the HU gradually slows down until it stops.
10. The water level in zones I, II, and III decreases to the downstream level, ensuring the HU is stationary.
11. Zones I, II, and III are then fully drained, completing the shutdown process. The downstream gate is closed.

### 3.2. Behavior of the Head During Operational Stages of the Hydraulic Unit

The hydroturbine head-cover experiences significant mechanical and hydraulic stresses throughout the operational stages of the HU. Table 1 provides a summary of the hydroturbine head-cover’s structural behavior during each operational stage.

As previously described, the hydroturbine head-cover experiences maximum deformation at the startup stage of HU operation. In contrast, during both partial- and full-load conditions, the deformation of the head-cover is reduced. However, it is crucial to identify the specific regions of the head-cover where maximum and minimum deformations occur. Additionally, understanding the extent to which head-cover deformation influences the deformation of its fastening bolts is essential for comprehensive analysis [19].

## 4. Vibrations and Deformations in Francis Turbines

Since additional stresses caused by vibrations are one of the contributing factors to the failure of the bolts that fasten the hydroturbine head-cover [12,13,14], it is essential to initially identify and comprehend the underlying causes of vibrations in the hydroturbine and their impact on the fastening bolts.

### 4.1. Vibrations in Hydroturbines: Causes, Consequences, and Mitigation Methods

#### 4.1.1. Causes and Consequences of Vibrations

According to the literature, accidents arise from mechanical and hydraulic vibrations, as well as fluctuations in electrical power [20,21]. Table 2 presents the factors of vibrations and their locations where they originate.

As a result of the factors presented in the table above, various types of mechanical vibrations occur in the hydroturbine. Article [30] mentions that shaft misalignment causes vibrations in both radial and axial directions. The authors of article [31] explain that increased vibrations in the hydroturbine result from bearing looseness and the imbalance of different components within the rotating assembly. When investigating the vibration characteristics of hydroturbines, the authors of [32] demonstrated that there are axial, radial, and torsional vibrations. Furthermore, they explain that axial vibrations result from the combination of the gravitational force of the hydraulic unit and axial hydraulic thrust. Table 3 summarizes the effects caused by vibrations on the hydroturbine head-cover and the bolts that fasten it.

#### 4.1.2. Methods for Mitigating Vibration

According to the literature, the following methods have been proposed to mitigate resonances and high vibration levels in HU:

- Minimizing mechanical imbalance, reducing electrical power fluctuations, decreasing stator/rotor eccentricity, and minimizing shape deviations in rotors and stators [36,37];
- Reducing vibrations caused by water flow [38,39];
- Optimizing design solutions for hydroelectric power plants and mechanical structures of HU components to prevent resonances and elevated vibration levels [40,41,42];
- Enhancing of damping and stiffness to dissipate vibrational energy, ensuring adequate stiffness of HU bearings to limit vibrational displacements, and employing radial dampers to reduce shaft vibrations at resonant frequencies [19,43];
- Enhancing mathematical models for hydroturbines and the application of reliability-based multidisciplinary design in turbine optimization [20,44];
- Improving vibration governance systems [32].

Thus, all these approaches are aimed at improving the design of HU components and ensuring their appropriate installation to decrease vibrations.

### 4.2. Mechanical Stresses and Deformations in a Hydroturbine

Sources that induce vibrations in hydroturbines not only generate vibrations but also create stresses and deformations within the structure of the hydraulic unit. The present article will focus on the mechanical stresses and deformations occurring in the fastening bolts of the hydroturbine head-cover during operation.

#### 4.2.1. Mechanical Stresses and Deformations in the Hydroturbine Head-Cover

**Figure 3:**
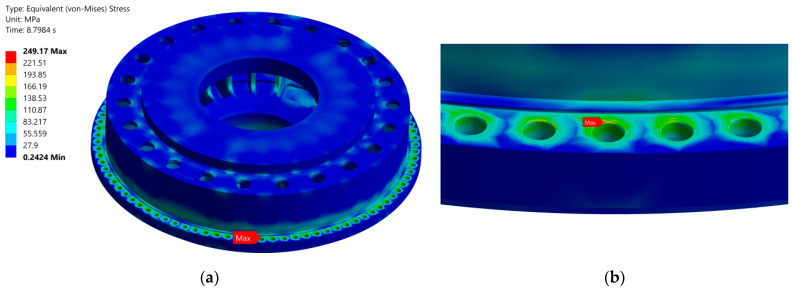
Von Mises stress in the hydroturbine [13]: (**a**) head-cover; (**b**) perimeter of the head-cover.

Research indicates that the mechanical stresses in the components of Francis turbines will be maximum at the head-cover perimeter, where the fastening bolts are located [13,19,45]. This is valid for both preloading (the initial stress exerted on the fastening bolts during tightening at installation) and after hydraulic pressure is applied (additional stresses caused by dynamic hydraulic forces acting on the hydroturbine during operation) [13]. The mechanical stresses in the hydroturbine head-cover are illustrated in Figure 3 using equivalent Von Mises stresses.

**Figure 4:**
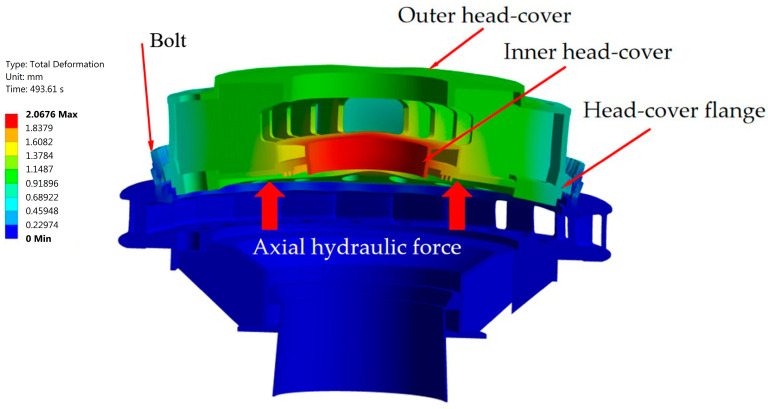
Deformation of hydroturbine head-cover with a single-flange plate [13].

On the other hand, the maximum deformation in the hydroturbine head-cover occurs around its axis during hydroturbine start-up, when the head-cover is lifted by axial hydraulic thrust (see Figure 4). In this process, the deformation at the outer edges, where the hydroturbine head-cover is fastened to its casing by bolts through a staying ring, will be minimal [13,46]. The described deformation is valid for head-covers with single- and double-flange plates.

Table 4 presents the deformation values in the hydroturbine head-cover, as reported in the studies [13,45].

#### 4.2.2. Mechanical Stresses and Deformations in the Fastening Bolts of Hydroturbine Head-Covers

**Figure 5:**
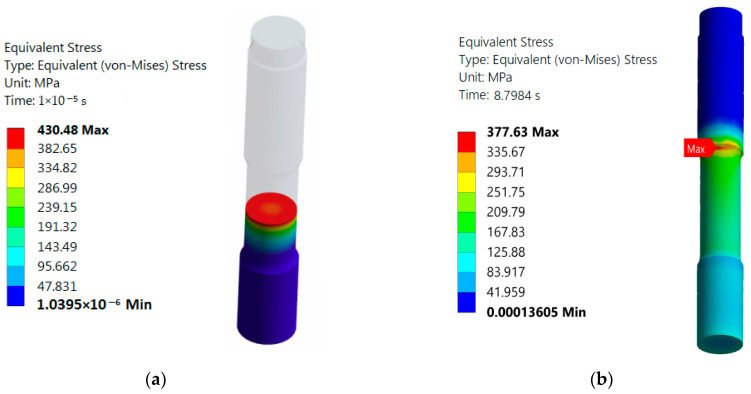
Distribution of Von Mises stresses on bolts [13]: (**a**) after installation; (**b**) during operation.

According to [13], the maximum mechanical stress is located in the central part of the fastening bolts after their installation in the hydroturbine head-cover (see Figure 5a). Results obtained from studies [38,46], which modeled deformations in the fastening bolts, indicate that during hydroturbine operation, the maximum mechanical stress shifts to the lower part of the bolt thread (see Figure 5b).

Furthermore, the deformation shape in the bolts is determined by the axial force acting on the hydroturbine head-cover, which pushes it upward, creating a tensile force that pulls the bolts along their axis. The authors of [13] demonstrate that the axial hydraulic thrust force lifts the head-cover with single-flange plate, resulting in radial outward bending of the bolt. In contrast, for a head-cover with a double-flange plate, the bolts bend and deform towards the weaker side of the flange due to the bending moment induced by the combined effects of preload and hydraulic thrust on the head-cover [32,47,48].

In contrast, studies have identified various types of deformations that occur in the hydroturbine head-cover and the bolts that fasten it to its casing, which vary according to the operational stage. These deformations are detailed below [13,19,49,50]:

- Elastic deformations are present throughout different operational phases, specifically during start-up, steady-state operation under partial and full loads, load transients, and shutdown. Elastic deformations arise from the gradual increase in water pressure and hydrodynamic forces. The hydroturbine head-cover is subjected to constant loads, including water pressure, centrifugal forces, and its own weight, which induce reversible elastic deformations. Consequently, similar elastic deformations also occur in the fastening bolts.
- Plastic deformations are associated with transient events such as water hammer, electrical failures, and load rejection. During these events, extreme pressure peaks, particularly those occurring in water hammer incidents, can surpass the elastic limit of the material, resulting in permanent deformations.
- Fatigue deformations appear under intense cyclic loading, which can accelerate the appearance of fatigue in the fastening bolts.

Under certain conditions, the following deformations may occur [44,51,52]:

- Torsional deformations may arise during the tightening process or under operational conditions where bolts are subjected to torsional loads. Specifically, during tightening, bolts experience torsional stress as a result of the applied torque. This stress is not typically a deformation issue but rather a factor in achieving the desired preload.
- Shear deformations can occur under specific conditions like fault slip or asymmetric hydraulic forces, particularly during transient events.
- Creep deformation is more likely to occur after extended periods of continuous operation, although its likelihood also depends on the material properties of the structures. Elevated component temperatures, resulting from friction or heat transfer from the water, can also accelerate creep deformation over time.

The analysis of deformation types is essential to determine whether the deformations caused by stresses that occur during the operation of a hydroturbine exceed the deformation limits (elastic, plastic, or others) of the materials used in its components, such as the hydroturbine head-cover, bolts, and nuts. Establishing acceptable deformation limits facilitates the development of monitoring systems, thereby enhancing the safety and effectiveness of the hydraulic unit’s operation [53].

## 5. Instruments for Vibration Measurement and Methods for Measuring Deformations in Francis Turbines

Enhancing the efficiency of electricity generation systems in hydroelectric power plants begins with mitigating negative impacts on their operation [54]. To ensure operational safety and smooth operation, various measuring instruments are employed to monitor static and dynamic characteristics, such as pressure pulsations, vibrations, and displacements in different parts of a hydraulic unit structure. Therefore, the present section is devoted to examining the methods and measuring instruments used for vibration and deformation monitoring in a hydraulic unit.

### 5.1. Instruments for Vibration Measurement

There are two primary groups of methods for measuring vibrations: those involving physical contact and those without physical contact. As indicated in publications [28,36] and the GOST R 70810-2023 standard [55], the following measuring instruments are utilized for vibration measurement: vibration sensors, proximity sensors (displacement sensors), velocimeters (velocity meters), and accelerometers. Each of these sensors can be based on various physical principles, including potentiometric, inductive, capacitive, electrical, and optical [30,36,56]. Table 5 provides a comprehensive overview of the measurement locations and directions associated with the measurand, along with the types and quantities of sensors utilized for monitoring the hydraulic unit.

### 5.2. Methods for Enhancing the Effectiveness of Vibration Monitoring Systems

To enhance the effectiveness of vibration monitoring systems, it is advisable to carry out the following:

- Consider the ISO 20816-5:2018 standard, which outlines methodologies for measuring various vibration parameters (displacement, velocity, and acceleration) and specifies the points and directions for measurements [57];
- Use the requirements of GOST R 70810-2023, which establishes guidelines for measuring vibration at support nodes (bearings) and criteria for interpreting measurement results, aiding in decision making regarding the need for equipment repair or maintenance [55];
- Perform periodic vibration analysis of the generator and hydroturbine to assess their condition and prevent potential issues [32,37];
- Simulate and predict changes in the vibration characteristics of the hydraulic unit shaft [58,59];
- Apply non-destructive testing methods [60,61,62,63], including the following:
  – Ultrasonic test and magnetic particle inspection to detect internal defects in bolts, evaluate mechanical stresses, and identify potential cracks or gaps [64,65,66].
  – Optical inspection of axially symmetric components to check the state of the fastening bolts [67];
- Utilize intelligent sensor networks (ISO/IEC 29182-4:2013) [68] and data analysis techniques to identify vibration-related problems and to predict equipment failures [69,70,71].

Together, these approaches aim to improve the reliability and safety of hydraulic units by optimizing monitoring and maintenance methods.

### 5.3. Methods for Measuring Mechanical Stress and Deformation

The deformation of the hydroturbine head-cover and fastening bolts is a critical factor in their operation. Studies of deformation modeling methods for hydroturbine structures do not sufficiently reflect the problems of measuring the deformation of the bolts that fasten the hydroturbine head-cover to its casing. Table 6 presents relevant information on simulation methods used for various analyses of hydroturbines, including mechanical stress and deformation. Additionally, it outlines both contact and non-contact measurement methods that can be employed to assess fastening bolt deformation.

## 6. Monitoring Systems for Hydroturbine Head-Cover Fastening Bolts at SSHPPs

In Section 4, a comprehensive review of studies on the modeling and analysis of vibrations and deformations in hydroturbine structures was conducted. The emphasis of the review was in the interaction of the fastening bolts and the head-cover. Additionally, different measuring instruments designed for monitoring vibrations and deformations were presented in Section 5. However, the available information on monitoring systems for the condition of fastening bolts connecting the head-cover and hydroturbine casing is insufficient. In searching for scientific information in the sources mentioned in Section 2, only two articles [78,79] were found on this topic.

The following provides brief descriptions of the fastening bolt monitoring systems that have been installed on Francis turbines at SSHPPs.

### 6.1. Mechanical Stress Monitoring System

The mechanical stress SKDS-SISH system is designed to measure the axial compressive forces acting on the bolts [80]. The operation principle of this system is based on converting the axial compressive force applied to the force-measuring washer into alterations in the resonance frequencies of radio signals reflected from passive acoustic–electronic sensitive elements installed in the washer [78]. The sensitive elements of the SKDS-SISH system convert the axial compressive force into a proportional change in the frequency of surface acoustic waves. This frequency change is proportional to the force applied to the washer, enabling the measurement of the force magnitude through the detection of frequency changes. Consequently, SKDS-SISH systems are employed to monitor the dynamic state of threaded connections, specifically the fastening bolts of the hydroturbine head-cover.

**Figure 6:**
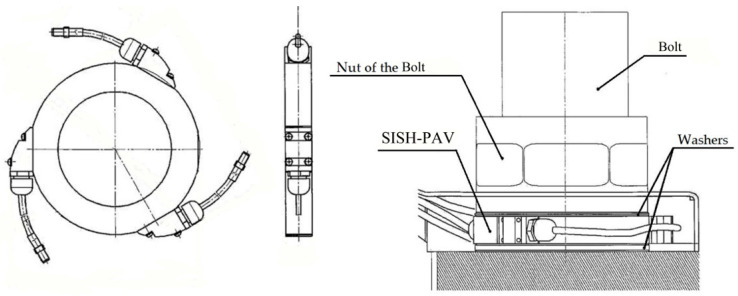
External view and scheme of installation of the SISH-PAV module on the bolt [81].

The SKDS-SISH system consists of eight modules of surface acoustic wave force-measuring washers (SISH-PAV). Each SISH-PAV module is equipped with three independent sensitive acoustic–electronic elements (see Figure 6).

Monitoring the metrological characteristics of measuring instruments helps prevent emergency situations by enabling the timely detection of malfunctions or deviations in equipment performance [82]. Therefore, Table 7 presents the metrological characteristics of the SKDS-SISH system.

### 6.2. Deformation Monitoring Systems

Two different systems designed for monitoring the deformation of fastening bolts connecting the hydroturbine head-cover to its casing are presented below.

The first system is KM-Delta-8-CM, which is intended for measuring linear deformations at the ends of the bolts relative to calibration rods installed within the bolts [83]. Each KM-Delta-8-CM system consists of eight modules for measuring linear displacements (MKLP), which contain linear displacement transducers LIR-DA 13B [84]. The metrological characteristics of this system are presented in Table 8.

The second system is PTK KM-Delta. As described in the documentation [85], this system is designed to measure the deformation of hydroturbine head-cover fastening bolts (linear displacement of bolt ends) and generate signals regarding hazardous and emergency deformations of these bolts.

The operational principle of PTK KM-Delta is similar to that of the KM-Delta-8-CM system. However, measurements are conducted using linear displacement transducers LIR-DA 13A [86], which are also installed in MKLP modules.

Both LIR-DA 13B and LIR-DA 13A are absolute optoelectronic displacement transducers designed to convert linear displacements of the controlled object into a digital code. This digital code represents the numerical equivalent of the displacement interval length, corresponding to the deformation of the bolt, relative to the origin defined by the calibration rod. The transducer features a measuring tip and operates on the principle of photoelectric detection, employing a contact-based measurement method.

**Figure 7:**
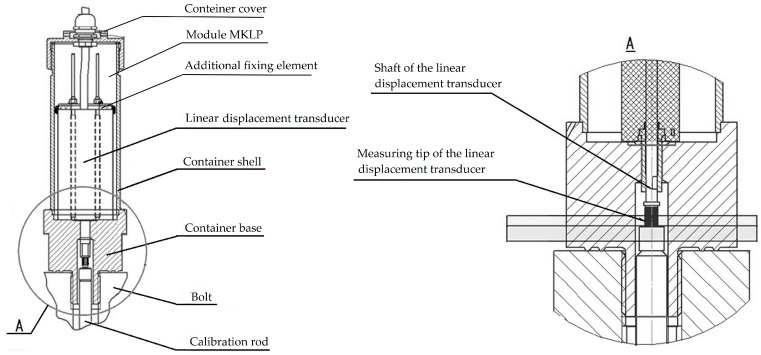
Scheme for mounting the MKLP module on the bolt [81].

In addition to the linear displacement transducer, the MKLP module comprises a container. Its main components include a shell, a cover, additional fixing elements, and a base (see Figure 7).

According to article [70], PTK KM-Delta replaced the previous version, KM-Delta-8-CM, for monitoring the deformation of fastening bolts on the hydroturbine head-cover at an SSHPP. The PTK KM-Delta is an intelligent multi-channel measurement system featuring a ‘metrological self-check’ function that extends its calibration interval from one year to four years. This function was developed in accordance with GOST R 8.673-2009 [87] and GOST 8.734-2015 [88]. The metrological characteristics of the system are presented in Table 9.

As shown in Table 8 and Table 9, the PTK KM-Delta system exhibits higher absolute error limits, indicating a lower precision in its measurements relative to the KM-Delta-8-CM system.

According to technical documents [83,85], data from each MKLP module is transmitted via the RS-422 interface for the KM-Delta-8-CM system and the RS-485 interface for the PTK KM-Delta system, utilizing SSIs (Silent Speech Interfaces) and Modbus RTU (Remote Terminal Unit) protocols. Measured data are subsequently processed and converted into values representing the displacement of the LIR-DA 13B or LIR-DA 13A transducer measuring tip. Finally, the processed information is transmitted through an Ethernet interface and displayed on the control panel mounted on the front panel of the control cabinet for each system.

According to [89], an SSI is ideal for point-to-point communication between a leader (e.g., controller) and a follower (e.g., sensor), making it suitable for precise measurements. On the other hand, the Modbus RTU protocol that operates under RS-485 is favored for its simplicity, reliability and cost-effectiveness [90].

A detailed description of the installation of the MKLP modules of the KM-Delta-8-CM system on the hydroturbine head-cover fastening bolts is not provided. However, the installation of the MKLP modules of the PTK KM-Delta system involves a specialized tightening procedure for the fastening bolts. The bolt is tightened with a nut until it reaches the elastic limit of the metal, specifically 40 × 13 steel. This controlled tightening induces the maximum elastic deformation of the bolt. Any subsequent deformation will be plastic, as the material will have exceeded its yield point. This process is designed to prevent the torsional deformation of the bolt. The calculated elongation of the fastening bolt after tightening is 490 µm. It should be clarified that this calculation is specific to the fastening bolts used in the SSHPP hydroturbine head-cover.

### 6.3. Deformations and Tensile Forces in the Hydroturbine Head-Cover Fastening Bolts

The measurement results of the fastening bolt deformation obtained using the PTK KM-Delta system in operation have not been published. Nonetheless, valuable insights into the deformation changes can be inferred from publication [81], where the testing of the fastening bolt deformation using the KM-Delta-8-CM system is discussed. This inference is rightful because both systems utilize linear displacement transducers, which are employed to measure the length and position of objects [83,85,91]. Additionally, in both cases, physical contact with the monitored object is required.

It is important to emphasize that the author of publication [82] does not provide numerical results for the deformation of the bolts. However, the presented results allow for a qualitative understanding of the deformation process.

**Figure 8:**
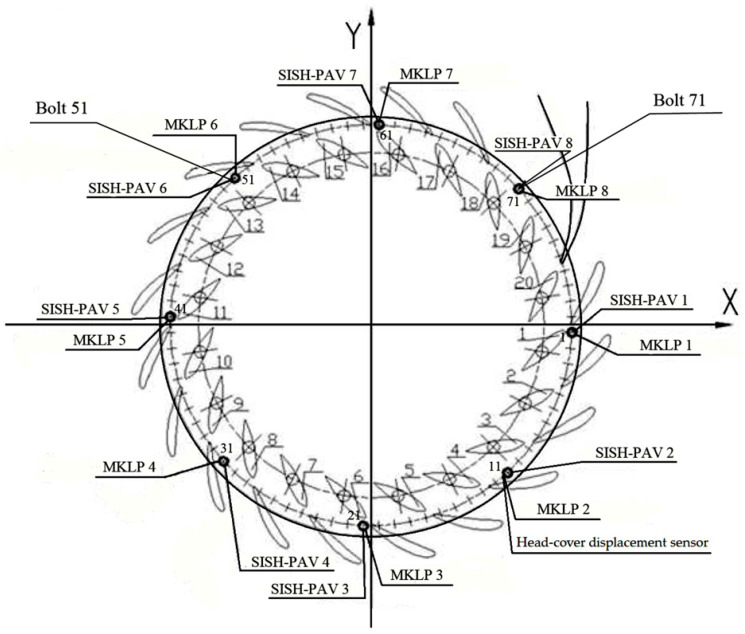
Scheme of placement of MKLP and SISH-PAV modules on bolts [81].

As explained in the aforementioned study [81], data were collected on a hydraulic unit to determine the elongation of the fastening bolts that secure the hydroturbine head-cover, as well as the forces acting on these bolts. For this purpose, eight MKLP modules and eight SISH-PAV modules from the KM-Delta-8-CM and SKDS-SISH systems were installed on every tenth bolt out of a total of 80 bolts located around the perimeter of the hydroturbine head-cover, as illustrated in Figure 8. It should be noted that in this study, the term ‘elongation’ refers to the ‘linear deformation’ of the fastening bolts.

The force measurement washers SISH-PAV measure the tensile force acting on the bolts, while the MKLP modules measure the elongation of the fastening bolts relative to calibration rods. It is important to note that tensile force and mechanical stress are interrelated through the deformation process, as mechanical stress is directly proportional to the relative elongation of the material under small deformations (Hooke’s Law) [92].

According to [81], the data collection was based on the following parameters:

- Head (the vertical difference in water levels between the upper and lower reservoirs).
- Displacement of the hydroturbine guide vanes using a guide device (GD), which regulates water flow to the runner for controlling turbine power.
- Water pressure in the spiral chamber.
- Vertical displacement of the hydroturbine head-cover.
- Elongation of the fastening bolts.
- Force exerted by the bolt on the hydroturbine head-cover.

Additionally, tests were conducted as follows:

- Before the flow section is drained.
- After its filling.
- During rotor unlocking.
- During the operation of the hydraulic unit in the electrical grid.

The results obtained from the tests indicate that during the operation, an interaction takes place between the static and dynamic forces of hydraulic pressure acting on the hydroturbine head-cover. This interaction leads to a change in the gap between the head-cover and the casing [81]. In other words, an increase in axial force is accompanied by the elongation of the fastening bolts around the perimeter of the hydroturbine head-cover.

**Figure 9:**
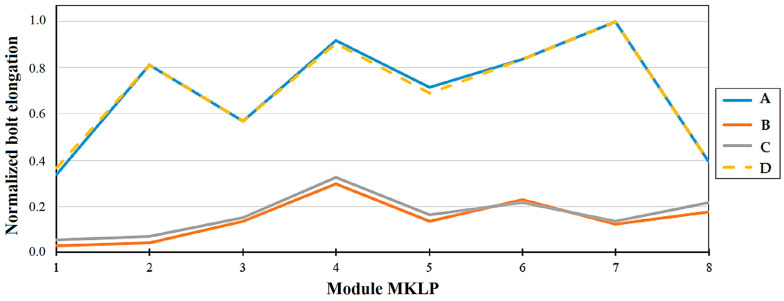
Trends in elongation of the bolts. The *x*-axis is the MKLP module number installed on the bolt; the *y*-axis is the normalized value of the elongation [81]. A—elongation of bolt before draining for H1 m; B—elongation of bolt after draining for H2 m; C—elongation of bolt before filling for H3 m; D—elongation of bolt after filling for H4 m.

**Figure 10:**
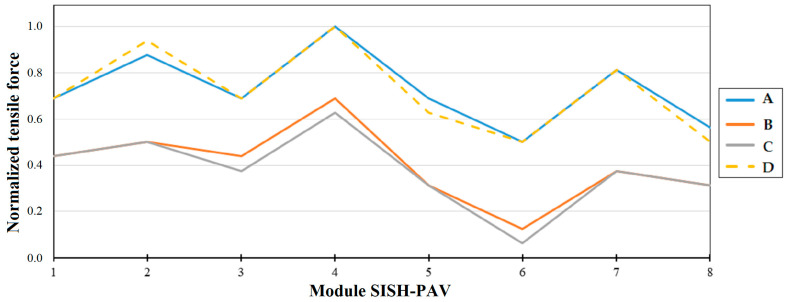
Trends in tensile force of the bolts. The *x*-axis is the SISH-PAV module number installed on the bolt; the *y*-axis is the normalized value of the tensile force [81]. A—tensile force before draining for H1 m; B—tensile force after draining for H2 m; C—tensile force before filling for H3 m; D—tensile force after filling for H4 m.

To investigate the elongation of the bolts and their load distribution, the author of [81] highlights the following trends in the condition. Figure 9 and Figure 10 present the normalized parameters of bolt elongation and tensile force in the bolts at different water head levels (H1, H2, H3, and H4).

As demonstrated in Figure 9, the elongation of the fastening bolts around the perimeter of the hydroturbine head-cover is not uniform. Measurement results obtained using the SKDS-SISH system indicate that the load experienced by the hydroturbine head-cover is also uneven. Furthermore, it is noted that the nature of the elongation of the bolts (Figure 9) is primarily similar to the pattern of tensile forces acting on the bolts, as shown in Figure 10.

**Figure 11:**
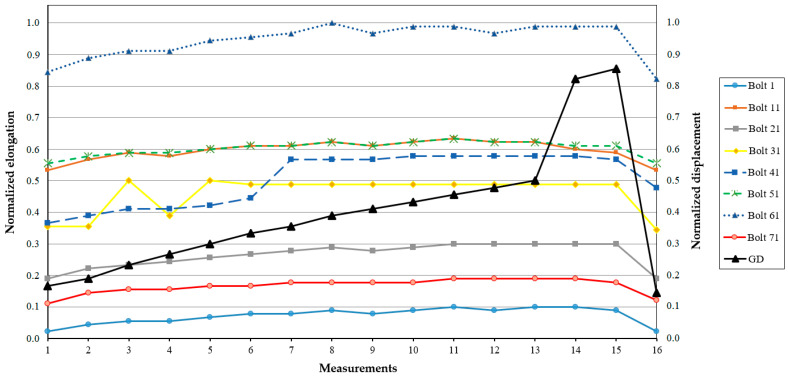
Measurement results of the bolt elongation [81]. *x*-axis—measurement number; *y*-axis (**left**)—normalized bolt elongation; *y*-axis (**right**)—normalized displacement of the GD.

**Figure 12:**
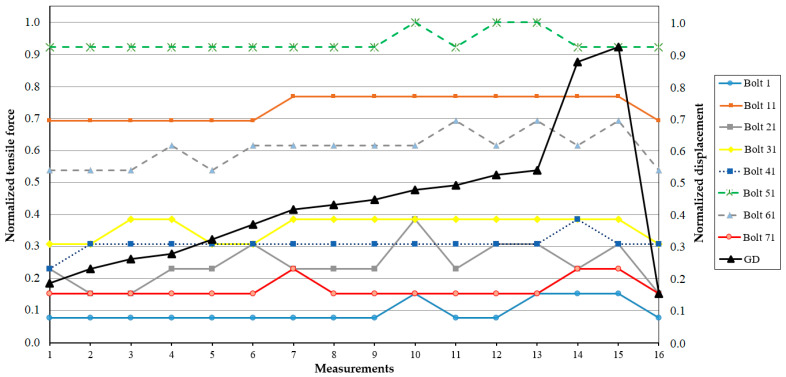
Measurement results of bolt tensile force [81]. *x*-axis—measurement number; *y*-axis (**left**)—normalized tensile force on the bolt; *y*-axis (**right**)—normalized displacement of the GD.

As reported in [81], subsequent tests on one of the hydraulic units during operation with increased power and displacement of the guiding device to open the vanes showed that the system behaves in a stable way, with no critical deviations observed. Figure 11 and Figure 12 show the normalized parameters of bolt elongation and tensile force in the bolts.

## 7. Analysis and Discussion

According to the theory [13,29,31], the axial force acting on the entire hydroturbine head-cover and pushing it upward creates significant deformation around its axis, while the deformation along the perimeter will be less pronounced. On the other hand, such an effect increases the mechanical stresses between the nuts installed on the fastening bolts and the hydroturbine head-cover. This means that the greater the axial hydraulic force that is converted into the elongation (linear deformation) of the bolts, the greater the mechanical stresses (tensile force) caused by the axial tensile forces.

The principle outlined above is not fully consistent with certain measurements obtained using the KM-Delta-8-CM and SKDS-SISH systems. As shown in Figure 9 and Figure 10, the tensile force measured with SISH-PAV module No. 7 is lower compared to the others, yet the deformation of the corresponding bolt is observed to be the largest. Another notable discrepancy is evident in the measurement results presented in Figure 11 and Figure 12, which pertain to one of the hydraulic units of the SSHPP. Specifically, the elongation of bolt No. 61 is found to be greater than that of bolt No. 51, despite the tensile force on bolt No. 51 being measured as higher than on bolt No. 61. These observations suggest that additional factors may be influencing the relationship between tensile force and bolt deformation. For instance, as discussed in [93], the stress behavior in metals exhibits distinct changes upon reaching the plastic deformation limit. Or in the worst case, the sensors are not working properly.

As discussed in Section 4.2, the maximum deformation of the hydroturbine head-cover occurs during the start-up stage of the hydraulic unit. This deformation is transmitted to the fastening bolts, causing them to deform as well. In addition, as noted in Section 6.2, the fastening bolts are tightened to the elastic limit of the metal, meaning that any additional deformation will be plastic. Consequently, the deformation incurred during start-up is not reversible and may accumulate between successive start-up cycles of the hydro unit. We consider this information relevant for maintaining records and establishing control tables that correlate fastening bolt deformations with the operational cycles of a hydraulic unit. Such records could facilitate further analysis and contribute to the development of fastening bolt deformation monitoring systems.

The results derived from the models developed in studies [46,47] reveal that the stresses in the fastening bolts installed around the perimeter of the hydroturbine head-cover are not uniform. Consequently, this suggests that the deformations of each fastening bolt will also differ based on their location. These findings are corroborated by the results obtained using the SKDS-SISH and KM-Delta-8-CM systems, as reported in study [82]. Specifically, the elongations (Figure 9 and Figure 11) and tensile forces (Figure 10 and Figure 12) measured for eight fastening bolts exhibit non-uniform behavior, further supporting the notion of location-dependent stress and deformation patterns. Additionally, the behavior appears to depend on the static and dynamic loads of hydraulic pressure on the hydroturbine casing [81], as well as on the tensile force experienced by the bolt when it is positioned closer to a particular supporting rib of the hydroturbine head-cover [13,32,46].

The KM-Delta-8-CM and PTK KM-Delta measurement systems were designed to assess the connection state of the hydroturbine head-cover to its casing through the continuous monitoring of the deformation of eight fastening bolts. The systems allow for simultaneous data collection from multiple locations, providing a more comprehensive understanding of the head-cover connection. These types of systems are known as multipoint monitoring systems and can help identify patterns or anomalies that may not be evident from single-point measurements. However, to ensure a secure connection between the hydro-turbine head-cover and its casing, it is crucial that all sensors at the measuring points function correctly throughout different operational stages of the hydraulic unit and maintain their performance over extended periods. The technical documentation [83,85] and publication for the KM-Delta-8-CM and PTK KM-Delta systems [79] do not provide evidence that this requirement is fulfilled. This implies that the sensors may fail during different operational stages of the hydraulic unit. Consequently, the trustworthiness of the systems cannot be guaranteed, and they are vulnerable to measurement errors.

Potential solutions to this problem can be realized through the implementation of novel approaches and/or the integration of emerging technologies, including the following:

- Self-check function—the implementation of a self-check function in measurement sensors enables the autonomous evaluation of their functionality and performance, eliminating the need for external intervention. Advanced sensors can perform metrological self-checks by comparing their measurements against built-in reference standards, thereby ensuring long-term accuracy and reliability [94]. In networked systems, self-checking sensors can transmit status alerts, facilitating proactive maintenance and preventing data corruption caused by malfunctioning sensors [95].
- Artificial Intelligence (AI) for sensor assurance—AI tools, such as neural networks and machine learning algorithms, generate reference models that capture ‘normal’ sensor behavior. These models enable the detection of deviations resulting from tampering or natural degradation [96]. Additionally, by distinguishing between normal drift patterns and abnormal deviations, AI facilitates sensor self-calibration, thereby reducing the necessity for manual recalibration and ensuring consistent measurement accuracy over time [97].
- Digital Twins—developing a virtual physical replica of the measurement sensor or simulating the process of fastening bolt deformation could accurately reflect and predict the behavior of their physical counterparts in real time. This approach enables the simulation of the measurement system’s behavior to estimate measurement uncertainty, allowing immediate adjustments and optimizations that ensure high-quality measurements and reduce the risk of accidents [98].

Furthermore, based on the theory reviewed in Section 3, Section 4 and Section 5 for the improvement or development of a deformation monitoring system for hydroturbine head-cover fastening bolts, the following should also be considered:

- The impact of vibrations on contact and non-contact measuring sensors due to the presence of axial and horizontal vibrations during the operational stages of the hydraulic unit;
- The deformations of the fastening bolts in the operating stages of the hydraulic unit due to fatigue deformation can occur before the maximum plastic deformation leads to the failure of the bolt.

## 8. Conclusions

This article investigates the current state of deformation monitoring systems for the fastening bolts of Francis turbine head-covers. It summarizes research conducted primarily over the past four years on this topic and provides a critical analysis of published results related to modeling and measuring the deformation of bolts that fasten the head-cover and casing of a hydroturbine.

The main conclusions and recommendations are as follows:

- This research supports, based on the results of practical tests published in SSHPPs, the theoretical models that describe the non-uniform behavior of deformations in the fastening bolts of the hydroturbine head-cover.
- The analysis of the information has shown that static and cyclic loads can lead to a gradual degradation of bolts over time, as well as induce fatigue, leading to bolt weakening. Therefore, the development of new deformation monitoring systems for fastening bolts must consider the cumulative damage resulting from both static and dynamic effects.
- As the analyzed deformation monitoring systems for fastening bolts, KM-Delta-8-CM and PTK KM-Delta, do not fully guarantee the reliability of the measurement sensors, it is necessary to develop improvements based on new approaches and technologies such as AI for ensuring the safety and increasing the operational uptime of the hydraulic unit.
- In developing a deformation monitoring system for the fastening bolts of a hydroturbine head-cover, it is essential to consider the effects of vibrations on the measuring sensors, as well as the bolt deformations that occur during various operational stages of the hydraulic unit.

## Figures and Tables

**Table 1:** Head-cover behavior across different operational stages of an HU [compiled by the authors].

Stage Operation	Hydraulic Loads	Mechanical Loads	Structural Behavior	Refs.
Pre-Startup (steps 1 to 3)	none	the tightening of the fastening bolts induces mechanical stress around the perimeter of the head-cover	head-cover remains undeformed	[13]
Startup (steps 4 to 6)	increasing water pressure acts on the head-cover as the turbine starts; impact forces may occur if the startup is rapid	initial vibrations; thermal stresses due to temperature changes	maximum head-cover deformation due to water pressure thrust	[13,16,18]
Partial Load (step 7)	non-uniform flow causes pressure pulsations in the had-cover; low-pressure zones can occur that generate cavitation	vibrations and asymmetric forces due to uneven flow distribution.	fatigue in the head-cover, joints, and bolts due to cyclic loads and vibrations; local deformations can appear (i.e., changes in the shape or structure) due to asymmetric forces or imbalances in load distribution	[16,17]
Full Load (step 7)	stable water pressure acts on the head-cover	minimal vibrations due to uniform flow; axial (downward) loads resulting from the combined effects of water pressure and suction forces exerted by the suction pipe	head-cover is subjected to constant and predictable loads, which reduces the risk of fatigue and deformation; head-cover deformation decreases due to axial loads	[16,17,18]
Shutdown (steps 8 to 11)	water pressure in the head-cover decreases; potential water hammer effects if the shutdown is abrupt	vibrations during deceleration of the rotor and change in the water flow	hydraulic and mechanical loads are reduced, allowing the head to return to its shape	[17]

**Table 2:** Vibration factors in Francis turbines [compiled by the authors].

Part of the Hydraulic Unit		Factors	Refs.
Vibration in rotating parts	Turbine runner	mechanical imbalance hydraulic imbalance misalignment cavitations turbine bearing instability rough zone operation improper lubrication of mechanical parts defective bearings cracked or chipped blades and shaft	[22,23,24,25]
	Rotor	* all of the above rotor rubs	[22,26,27]
Vibration in non-rotating parts	Draft tube	cavitations power swings draft tube resonance	[22,24,25]
	Shaft seals	abrasive erosion (depends on water quality)	[28]
	Penstock	cavitations	[22,28]
	Generator	electromagnetic force	[22,26]
	Transformer	magneto motive forces	[22,28,29]

* Consider all factors occurring in the turbine runner.

**Table 3:** Vibration characteristics and their effects on the hydroturbine head-cover and its fastening bolts [compiled by the authors].

Stage Operation	Values and Orientation of Vibrations *	Effects on Head-Cover	Effects on Fastening Bolts	Refs.
Start-up	0.1 to 10 Hz axial and horizontal	Transient hydraulic forces cause sudden pressure changes, leading to stress on the head-cover. Vibrations may cause temporary misalignment.	Bolts experience initial stress due to uneven load distribution. Risk of loosening due to transient vibrations.	[33]
Partial load	10 to 50 Hz horizontal	Unsteady hydraulic forces (e.g., vortex ropes) cause cyclic loading and vibrations. Increased risk of fatigue damage.	Cyclic loading leads to fatigue in bolts. Uneven load distribution may cause some bolts to bear more stress.	[33,34]
Full load	50 to 100 Hz axial	High hydraulic and mechanical loads cause significant stress on the head-cover. Vibrations may lead to long-term deformation.	Bolts are subjected to high static and dynamic loads. Risk of creep and plastic deformation over time.	[34,35]

* General description of typical vibration values and their orientation.

**Table 4:** Values of axial deformations in the hydroturbine head-covers [compiled by the authors].

Hydroturbine	Water Head, m	Output Power, MW	Flow Rate, m^3^/s	Rated Speed, rpm	Deformation of the Hydroturbine Head-Cover Around the		Ref.
					Shaft, mm	Perimeter, mm	
–	340.0	300.0	–	–	1.84–2.06	0.23–0.45	[13]
TTS332	256.3	41.2	17.59	500	0.43	0.12	[45]

**Table 5:** Measurement locations, directions, and sensor specifications for hydraulic unit monitoring (compiled by the authors based on [28,55]).

N°	Measurement Locations	Measurement Directions	Measurand	Sensor
1	Upper rack	horizontal	absolute vibration	velocity
		vertical		
2	Stator core	horizontal	absolute vibration	acceleration
		vertical		
3	Lower rack	horizontal	absolute vibration	velocity
		vertical		
4	Head-cover	horizontal	absolute vibration	velocity
		vertical		
		axial		
5	Upper generator bearing	radial vibration	relative vibration	displacement
6	Lower generator bearing			
7	Shaft turbine	radial vibration	relative vibration	
8		—	displacement	
9	Servomotor of the guide vane	—	position	position
10	Spiral turbine casing	—	water flow	differential pressure
11	Under the turbine head-cover	—	pressure pulsation	pressure
12	Draft tube	—		
13	Spiral case	—		

**Table 6:** Methods that can be applied for the determination of mechanical stress and deformation of fastening bolts of hydroturbine head-covers [compiled by the authors].

Method	Technology	Application	Refs.
Numerical simulation methods	Computational Fluid Dynamics (CFD)	CFD is employed to carry out the following: Simulate fluid flow. Simulate pressure distribution on the head-cover and the fastening bolts during start-up and normal operation of the hydroturbine. Predict the performance of hydroturbines under different operating conditions. Analyze and understand cavitation phenomena.	[13,27,38,47,72]
	Finite Element Analysis (FEA)	FEA complements CFD by focusing on the following: Structural integrity of turbine components under operational loads; Mechanical stress and deformation assessment; Modal analysis (vibration frequency); Material and structure optimization.	[13,19,27,46,73]
Contact measurement methods	Strain gauge	This is used to measure the following: Axial forces acting on the hydroturbine; Dynamic stresses in hydroturbine structures; Fatigue of hydroturbine components.	[74,75]
	Surface acoustic wave (SAW) sensors	These are used to measure pressure and strain in rotating machinery.	[76,77]
Non-contact measurement methods	Eddy current sensors	These are used to measure the following: Movement and position of electrically conductive components; Deformation of the hydroturbine head-cover.	[45]

**Table 7:** Metrological characteristics of the SKDS-SISH system (compiled by the authors based on [80]).

SKDS-SISH System					
Number of measuring channels	Measuring range of compression force displacement, MN	Limits of absolute error, MN	Mass of SISH-PAV, kg	Probability of failure-free operation in 2000 h	Average lifespan, years
8	0.34 to 1.27	±0.04	1.5	≥0.9	10

**Table 8:** Metrological characteristics of the KM-Delta-8-CM system (compiled by the authors based on [83,84]).

KM-Delta-8-CM System				
Number of measuring channels	Full measuring range of displacement MKLP, µm	Limits of absolute error, µm	Mass of MKLP, kg	Scale division, µm
8	0 to 10,000	±5	2.3	1
Probability of failure-free operation in 30,000 h	0.98	Average lifespan, years	10	
**LIR-DA 13B Displacement Transducer**				
Measuring range of displacement, mm	Limits of absolute error, µm	Measuring force, N	Mass (without cable), kg	
0 to 10	±1.5	2.0	0.3	

**Table 9:** Metrological characteristics of the PTK KM-Delta system (compiled by the authors based on [85,86]).

PTK KM-Delta System							
Number of measuring channels	MKLP module displacement measuring range, µm			Limits of absolute error, µm	Mass of MKLP, kg	Scale division, µm	
	Full Range	During Mounting	In Operation				
8	0 to 10,000	5100 to 8300	250 to 760 *	±10	2.5	0.5	
Average MTBF, h		80,000	Average life-span, years		10		
**LIR-DA 13A Displacement Transducer**							
Measuring range of displacement, mm		Limits of absolute error, µm	Measuring force, N		Mass (without cable), kg		
−5 to 5		±5	≤1.5		0.25		

* After installation, the displacement value of MKLP module is conventionally set to 490 µm.

## Data Availability

Not applicable.

## Abbreviations

HPP	Hydroelectric Power Plant
IHA	International Hydropower Association
IEA	International Energy Agency
SSHPP	Sayano-Shushenskaya Hydroelectric Power Plant
HU	Hydraulic Unit
CFD	Computational Fluid Dynamics
FEA	Finite Element Analysis
SAW	Surface-Active Wave
SSI	Silent Speech Interface
RTU	Remote Terminal Unit
SISH-PAV	Surface Acoustic Wave Force-measuring Washer
MKLP	Measuring Linear Displacement
GD	Guide Device
AI	Artificial Intelligence
